# Recent Progress in Metabolic Signaling Pathways Regulating Aging and Life Span

**DOI:** 10.1093/gerona/glu058

**Published:** 2014-05-08

**Authors:** Christopher B. Newgard, Jeffrey E. Pessin

**Affiliations:** ^1^Sarah W. Stedman Nutrition and Metabolism Center and Duke Molecular Physiology Institute, Duke University Medical Center, Durham, North Carolina.; ^2^Department of Medicine and Molecular Pharmacology, Albert Einstein College of Medicine, Bronx, New York.

**Keywords:** Signal transduction, Intermediary metabolism, Metabolic pathways.

## Abstract

The NIH Summit, Advances in Geroscience: Impact on Health Span and Chronic Disease, discusses several aspects of cellular degeneration that underlie susceptibility to chronic aging-associated diseases, morbidity, and mortality. In particular, the session on Metabolism focuses on the interrelationship between signal transduction, intermediary metabolism, and metabolic products and byproducts that contribute to pathophysiologic phenotypes and detrimental effects that occur during the aging process, thus leading to susceptibility to disease. Although it is well established that many metabolic pathways (ie, oxidative phosphorylation, insulin-stimulated glucose uptake) decline with age, it often remains uncertain if these are a cause or consequence of the aging process. Moreover, the mechanisms accounting for the decline in metabolic function remain enigmatic. Several novel and unexpected concepts are emerging that will help to define the roles of altered metabolic control in the degenerative mechanisms of aging. This brief review summarizes several of the topics to be discussed in the metabolism of aging session (http://www.geron.org/About%20Us/nih-geroscience-summit).

## Peripheral Tissue Metabolism and Signaling

Living entities, whether individual cells or multicellular organisms, utilize substrates and nutrients to fuel energy production, macromolecular synthesis/degradation, growth, and reproduction. As cells/organisms age, these biological processes become increasingly less efficient, and with time, cells and tissues become damaged as a result of these inefficiencies. The problem is further augmented by decreased ability to prevent and/or repair accumulating damage. For example, inefficient mitochondrial substrate oxidation increases generation of free radical species that in turn oxidize mitochondrial DNA. However, as mitophagy (macroautophagy of mitochondria) declines with aging, mitochondrial repair/renewal is also compromised.

Core metabolic pathways such as glycolysis, fatty acid oxidation, amino acid oxidation, lipogenesis, and ketogenesis are also altered in aging, and better insights into how this occurs is central to gaining a more comprehensive understanding of the forces that shape aging biology, the susceptibility to chronic diseases, and the interrelationship between the two. In particular, caloric restriction is well established to increase longevity and to improve aging-dependent phenotypes and is the classic paradigm for studying the role of metabolism in aging. This session will focus on molecular mechanisms and consequences of the aging process, several of which have been elucidated through studies of caloric restriction.

Two critical enzyme complexes that have received attention in control of aging are the nutrient and hormone sensing mammalian target of rapamycin complex 1 (mTORC1) and the AMP-dependent protein kinase (AMPK). These two enzymes are reciprocally regulated, in that AMPK is activated during nutrient deprivation and low energy charge, whereas mTORC1 is activated by growth factors and in states of nutrient abundance (1). AMPK activation primarily drives catabolic processes that yield ATP and restore cellular energy state, whereas mTORC1 drives anabolic process to increase energy storage and macromolecular biosynthesis. Evidence that these pathways are intimately involved in the aging process is built largely from studies on the effects of rapamycin (mTORC1 inhibitor) and metformin (an indirect AMPK activator).

Rapamycin was originally identified as an immunosuppressive drug used to prevent organ rejection and more recently as a tumor growth inhibitor. It displays longevity properties in several genetic systems including *Saccharomyces* *cerevisiae*, *Drosophila* *Melanogaster*, *Caenorhabditis* *elegans*, and mice (2). Although mechanisms underlying rapamycin-induced increases in life span have not been fully elucidated, there are several intriguing clues. One major function of mTORC1 is the stimulation of protein synthesis (~30% of total protein synthesis is mTORC1 dependent) through the regulation of S6 kinases 1 and 2 and the 4E-binding proteins E4-BP1 and E4-BP2 (3). Thus, rapamycin inhibition of mTORC1 may promote survival by reducing protein synthesis and thereby relieving endoplasmic reticulum stress, which can drive a variety of detrimental cellular responses.

Autophagy is an important catabolic process that mediates degradation of intracellular components (proteins and organelles) in lysosomes and has also been shown to contribute to maintenance of cellular energetic balance (4). There are three forms of autophagy (macroautophagy, microautophagy, and chaperone-mediated autophagy) with macroautophagy being the most intensively investigated. Macroautophagy is characterized by de novo formation of double membrane phagophore structures that sequester organelles and long-lived proteins and subsequently generate large limited membrane vesicles termed autophagosomes that fuse with lysosomes, resulting in the degradation of the autophagosome contents. It is well established in multiple systems that macroautophagy declines with aging, whereas its restoration prevents several age-related pathologies. The mTORC1 complex inhibits macroautophagy through the phosphorylation and inactivation of ULK1, a critical initiating kinase of phagophore formation (2). Rapamycin suppression of mTORC1 increases macroautophagy initiation, serving as a possible molecular signal for maintaining this important cell survival pathway.

Metformin is thought to activate AMPK in part through inhibition of complex 1 of the mitochondrial electron transport chain, thereby increasing cellular AMP/ADP ratio (5). Although metformin is currently best recognized as the world’s most widely prescribed diabetes medicine, several studies have shown that long-term metformin treatment increases longevity in several species and decreases overall mortality in humans (6). AMPK has a variety of cellular functions. Among them, AMPK activation suppresses mTORC1 by inhibiting the mTORC1 subunit raptor and by activation of the Rheb GTPase TSC1/2 (7,8). In addition, AMPK also activates macroautophagy, not only through inhibition of mTORC1 but also by activating the ULK1 kinase (9–13). These findings provide an intriguing link between nutrient sensing and signaling pathways that may potentially account for the ability of rapamycin and metformin to promote longevity.

These studies leave an unanswered question—why do molecular signals that slow nutrient storage and enhance fuel oxidation increase longevity? One important issue is the use of the terms “longevity” and “aging.” In many cases, longevity and aging are used as synonymous terms, but in reality, longevity refers to the average life span, whereas aging is defined by the appearance of a set of aging-induced phenotypes that may or may not affect life span, including reduced fertility, frailty, loss of hearing and eyesight, and changes in hair/fur color. This is an important distinction as rapamycin has been routinely reported to increase longevity. However, a recent work suggests that rapamycin primarily increases longevity by preventing the development of disease pathologies and that most aging-dependent phenotypes are unaffected (14,15). Thus, further studies are needed to determine whether enhanced nutrient catabolism modulates longevity by reducing death-inducing pathologies (eg, cardiovascular disease, cancer, etc.) and/or reduces aging-dependent phenotypes associated with extension of healthy life span.

## Nicotinamide Adenine Dinucleotide Metabolism

Nicotinamide adenine dinucleotide (NAD) metabolism is also strongly related to healthy life span and longevity. NAD acts a coenzyme in redox reactions to form NADH, which can in turn serve as a reducing agent to donate electrons. In addition, NAD functions as a donor of ADP-ribose for the PARP family of poly ADP-ribose polymerases that ADP-ribosylate damaged DNA to repair single-strand DNA nicks (16). Interestingly, PARP activity is positively correlated with life span in several different species (17). Moreover, EBV-transformed lymphoblasts from centenarians displayed a 1.6-fold increase in PARP specific enzymatic activity compared with adult (20–70 years old) EBV-transformed lymphoblasts (18), suggesting that DNA repair capacity may be an important component defining the aging process. The role of DNA damage and repair will be discussed in the Macromolecular Damage session and is directly integrated with metabolic control discussed in the Metabolism session.

NAD also plays a role as an important cosubstrate for the sirtuin family of NAD-dependent protein deacetylases (now recognized more accurately as a family of protein deacylases), some of which appear to have longevity promoting properties. In mammals, there are seven members of the sirtuin family that are selectively localized to the cytosol, mitochondria, and nucleus. This family of enzymes removes acetyl and other acyl groups from a wide variety of proteins involved in transcriptional control as well as in metabolic regulation. An important connection between mammalian aging and sirtuin function is based upon the reduced activity of some sirtuins during aging, and the finding that caloric restriction, an intervention that promotes longevity, induces and prevents the aging-dependent decline in Sirt1 (nuclear) and Sirt3 (mitochondrial) protein levels. Similarly, elevation of NAD levels by nicotinamide mononucleotide administration to increase NAD synthesis (19), inhibition of PARP activity to prevent NAD depletion (20), or inhibition of the NAD glycohydrolase, CD38 (21,22) protects against diet-induced obesity, liver steatosis, and the metabolic syndrome.

Studies of sirtuin function and aging in humans have so far depended on correlative observations, and further insight will require safe development of pharmacological sirtuin activators. One potential sirtuin activator (Sirt1) is resveratrol. Treatment of mice with this compound results in increased mitochondrial biogenesis, AMPK activation, and increased NAD levels. Although several studies have reported increased life span in multiple species including yeast, worms, flies, and fish (23–26), it does not appear to alter life span in mice fed a normal diet (27). However, resveratrol was found to increase longevity in mice fed a high fat diet (28). The basis for the apparent dietary-dependent effects of resveratrol are not known but may reflect the ability of resveratrol to improve the metabolic profile of high fat diet fed mice (29).

In nonhuman primates, resveratrol was shown to improve adipose tissue insulin signaling and to reduce diet-induced inflammation (30). Similarly, in humans, resveratrol was reported to improve insulin sensitivity and postprandial glucose tolerance in older individuals and to augment the management of some human cancers (31,32). However, obese human subjects treated with resveratrol for 4 weeks had no significant improvement in hepatic glucose production, blood pressure, resting energy expenditure, lipid oxidation, fat content, or inflammatory markers (33). Similarly, resveratrol had no beneficial effect in overweight or obese men diagnosed with nonalcoholic fatty liver disease (34). Complicating these findings are reports that have questioned the specificity of resveratrol as a Sirt1 activator and have suggested that this natural product can have several other biological targets including activation of AMPK and cAMP-phosphodiesterase (35–37). Thus, the clinical use of resveratrol to improve metabolism and particularly healthy aging still remains an open question. Current studies are focused on development of more selective sirtuin activators for potential use in human studies (38).

In general, caloric restriction is the most effective maneuver for increasing healthy longevity in most eukaryotic species. However, isocaloric carbohydrate or isocaloric lipid restriction did not cause a significant increase in rodent life span, whereas isocaloric protein restriction was approximately 50% as effective in increasing longevity as general caloric restriction (39,40). More recently, the effect of protein restriction to increase longevity was found to be associated with decreased mTORC1 activation consistent with the life span promoting effect of rapamycin (41). In addition, multiple studies have shown that methionine restriction in an otherwise isocaloric diet increases rodent life span similar to protein restriction (42–45). Proposed mechanisms to explain the effects of methionine restriction on healthy aging include decreased reactive oxygen species (ROS) generation, reduction in cysteine levels, changes in gene expression, and reduction in *S*-adenosylmethyonine and *S*-adenosylhomocysteine levels (46).

## Metabolic Futile Cycling and Aging

An understudied aspect of metabolism is the physiologic function of futile cycles, a process where two metabolic pathways run in opposite directions resulting in the dissipation of energy in the form of heat. It is highly unlikely that futile cycles were designed in nature as continuous heat generators, suggesting that they may have other physiologic purposes.

One metabolic futile cycle that may potentially be involved in the aging process is the glycerol lipid/free fatty acid cycle. All eukaryotic cells store energy in the form of triacylglycerol (TG) and in mammals TG is predominantly localized to adipocytes. In states of energy deprivation, adipocyte-derived TG is hydrolyzed to free fatty acids and glycerol for release into the circulation. The fatty acids are used for cellular β-oxidation to generate ATP and glycerol is converted back to glucose via gluconeogenesis. In nutrient replete conditions, fatty acyl CoA is esterified to a glycerol-3-phosphate backbone to regenerate TG for storage. This cycle of TG hydrolysis and synthesis is a continuous process, and a large percentage of fatty acids are re-esterified back into TG. One intriguing possible connection between this cycle and longevity is the generation of NAD+ by glycerol-3-phosphate dehydrogenase (47). This cycle may help to maintain levels of NAD necessary for normal function of PARP and sirtuins that may be critical for the maintenance of important antiaging functions such as DNA damage repair and protein deacylation. This may also help to explain why activation of lipolysis and lipid oxidation by drugs that stimulate AMPK or inhibit mTOR contributes to the antiaging effect of such agents.

The reoxidation of NAD from NADH primarily occurs in the mitochondrial electron transport chain under aerobic conditions. However, the mitochondrial inner membrane is impermeable to NAD and NADH, and thus NADH shuttle systems are needed to transport NAD and NADH across the mitochondrial membrane, to equilibrate the NAD/NADH ratio between the mitochondria and the cytosol/nuclear pools. In yeast, the mitochondrial components of the malate-aspartate as well as other NAD/NADH shuttles appear to play an important role life span extension, dependent upon both mitochondrial respiration and sirtuin functions (48).

Another form of futile cycling of relevance to the aging process is the uncoupling of mitochondria. Oxidative phosphorylation creates a proton gradient that is used to generate ATP, but this process is incompletely coupled and redox energy can be lost due to the leak of protons across the inner mitochondrial membrane as heat. This proton leak may account for approximately 20% of resting mitochondrial respiration (49–51). A long-standing hypothesis of the aging field is that ROS production and associated cell damage can lead to the development of aging-like metabolic characteristics (52–54). However, the species of ROS and the subcellular location of antioxidant activity, rather than simply ROS levels, can strongly affect whether increased ROS production correlates with decreased life span (55). H_2_O_2_ production appears to be more directly related to life span determination than −O_2_ (superoxide), and a general hypothesis for uncoupling is that it may blunt membrane potential fluctuation to minimize ROS/H_2_O_2_ production.

Metformin has complex effects on longevity, and differences for its in vivo versus in vitro effects on the electron transport chain that may not be related to AMPK activation. As recently reported, chronic low dose daily metformin treatment activated AMPK without affecting electron transport chain activities in mice, whereas mouse embryo fibroblasts treated with metformin activated AMPK and decreased electron transport chain activities (56). In vivo, metformin treatment also induced Nrf2 target gene activation, a transcription factor that regulates the expression of multiple antioxidant genes and is also required for the beneficial effects of metformin in *C* *elegans* (57). Low dose of metformin increased life span, energy expenditure and decreased respiratory exchange rate, implying increased β-oxidation of fatty acids. However, a 10-fold higher dose of metformin was toxic, decreasing life span. Thus, the potential role of AMPK-regulated increases in β-oxidation and ROS production in aging phenotypes will require further critical evaluation.

## Circadian Rhythms and Aging

All mammalian cells have an intrinsic clock cycle that is regulated by specific clock genes. Most of the clock genes are transcription factors that activate or repress their own expression as well as that of other genes to create a self-sustaining transcriptional loop (58,59). Changes in expression, localization, and modifications coupled with specific temporal delays between transcription and translation lead to the approximate 24-h circadian cycle. Almost all tested tissues display circadian rhythms in gene expression, suggesting the existence of circadian clocks in most peripheral tissues. The central clock is located in the suprachiasmatic nucleus in the hypothalamus. The suprachiasmatic nucleus functions to maintain synchrony of the individual cellular circadian oscillators throughout the body. In mammals, these central cyclic rhythms are also modified (entrained) by local environmental cues such as the light/dark cycle. Several metabolic processes are influenced by the normal circadian rhythms including lipid and glucose homeostasis, mediated at least partially through control of lipogenic and gluconeogenic gene expression (60,61).

Epidemiology studies have also suggested an important role of the circadian clock in human pathophysiology. For example, important cardiometabolic disease events such as myocardial infarction and hypertensive crises occur more frequently at specific times of the day (62–64), and shift work increases the risk of development of cardiovascular and metabolic syndromes. Recent studies have also shown that circadian rhythms are altered during the aging process (65). In animal models, genetic disruption of circadian clock leads to reduced life span and accelerated development of age-associated pathology. The most severe example occurs in mice deficient for transcriptional factor BMAL1, which develop premature aging phenotype. Moreover, in a transgenic model of reduced food intake and longevity, there is greater expression of biological clock genes with increased amplitude and/or phase of the clock output systems (66–68). Together there is a strong correlation of robust clock cycling with longevity, while disrupted clock is associated with reduced life expectancy. Interestingly, a recent study demonstrates that clock genes regulate cycles of ATP as well as NAD production, which in turn serve to modulate mitochondrial protein acylation and synchronization of oxidative metabolic pathways (69).

Another important link between the circadian clock, aging, and metabolism is Sirt1. Sirt1 binding and deacetylase activity is regulated in a circadian manner through circadian oscillation of NAD production that in turn regulates the transcriptional expression of several key clock genes (70,71). Moreover, genetic Sirt1 deficiency or pharmacological activation alters circadian rhythm-regulated gene transcription (72,73). These and related studies suggest that Sirt1 functions as an important modulator of clock-mediated deacetylase activity, which in turn participates in control of the timing of histone acetylation and induction of transcription factors that control circadian physiology.

Sirt1 expression decreases in aged mice in concert with an increase in the normal length of the circadian cycle and an aging-associated inability to adapt to changes in external circadian cycle cues (65). Importantly, increasing Sirt1 expression allows older mice to display a similar adaptability of the circadian cycle as young mice. The apparent association of circadian clock dysfunction with age-associated disease, and the interaction of these pathways with NAD synthesis, sirtuin activity, and metabolic function provides further support for the development of more specific sirtuin and circadian clock activators for testing in various animal model systems to improve metabolic function, health and longevity. However, whether these processes are as intimately connected in human physiology as they appear to be in rodents and other animal model organisms, remains to be resolved.

## Hypothalamic Signaling

It is well established that several hypothalamic neural networks control feeding behavior, energy balance, and hepatic gluconeogenesis. Pro-opiomelanocortin neurons suppress appetite, whereas Agouti-related peptide (AgRP) neurons enhance food consumption (74,75). Interaction of these neurons forms a negative feedback loop where AgRP neurons suppress pro-opiomelanocortin neuron firing. The integration of these signals is controlled by a variety of circulating factors including leptin, insulin, glucose, fatty acids, and branched-chain amino acids.

Several studies have also suggested that the brain contributes to whole animal life span. For example, insulin receptor knockout mice have metabolic abnormalities resulting in neonatal death due to diabetic ketoacidosis (76,77). Re-expression of the insulin receptor in the brain, liver, and pancreatic beta cells is sufficient to normalize life span and to prevent diabetes, whereas mice with insulin receptor expression in non-brain tissues are protected against neonatal death but still die prematurely due to abnormal metabolism resulting from lipoatrophic diabetes. IRS2 (insulin receptor substrate 2) is a major proximal target of the insulin receptor. In contrast to the insulin receptor, whole body or brain-specific IRS2 knockout mice display an 18% increase in life span (78). More recently, the hypothalamus was found to be an important regulator of longevity in mice, and inhibition of hypothalamic NF-κB activation resulted in life span extension (79). Although the specific neural circuits responsible have not been defined, these data provide evidence for an important role of central signaling in life span that needs further exploration.

More recently, several studies have reported a complex integration between hypothalamic metabolism, inflammation, neural stem cell regeneration, and life span. Diet-induced obesity and insulin resistance is associated with hypothalamic endoplasmic reticulum stress and activation of the NF-κB inflammatory signaling pathway, and suppression of NF-κB signaling protects mice from diet-induced insulin resistance and endoplasmic reticulum stress (80). In parallel, NF-κB activation was found to suppress gonadotropin-releasing hormone (GnRH) expression. The loss of GnRH expression was found to be directly responsible for aging-impaired neurogenesis and was also associated with poor peripheral metabolic regulation (79).

## Summary

As evident from the above brief discussion, there is a close and intimate relationship between metabolic function and its regulation by key signaling pathways in control of aging and age-induced pathophysiology. However, whether aging induces metabolic dysregulation or metabolic dysfunction contributes to the aging process remains a difficult question that is not easily resolved. Current evidence suggests that improved metabolic efficiency with reduced nutrient storage and enhanced fuel oxidation serves to combat age-related disease processes, resulting in enhanced healthy longevity.

In the realm of metabolism, several pathways are deserving of further investigation for promoting increased health span. Current studies are beginning to shed light on the cellular/tissue targets, signals, and metabolic pathways relevant to the aging process, and those briefly highlighted in this overview will hopefully provide a fertile background for discussion at the upcoming Summit. In particular, we hope to address the following questions related to age-dependent nutritional requirements and metabolic control during the aging process.

1. How do signaling pathways that regulate growth and metabolism contribute to aging? Are these processes directly or indirectly related to age-associated chronic pathologies such as diabetes and cardiovascular diseases?
2. What are the molecular mechanisms that account for increased longevity resulting from caloric restriction and enhanced fuel oxidation? Is this due to a decrease in the age-induced phenotypes or simply an increase in longevity without improvements in specific aging parameters?
3. What is the role, if any, for futile metabolic cycles in regulating the aging process? Does aging-induced alteration in metabolite futile cycling create a state of altered metabolism and does this contribute to chronic disease incidence and progression?
4. Are the metabolic dysfunctions that occur during aging a result of cell autonomous defects or are they due to a CNS-mediated integrated response? Is there a relationship between hypothalamic degeneration and dysregulation of circadian control of metabolism?
5. Is the disruption of the normal clock cycle a cause or consequence of aging and what is the relationship between circadian rhythms and aging-induced phenotypes? Does altered sleep quality in aging contribute to dysregulation of circadian control of metabolism?

## Funding

This article was funded by National Institutes of Health (DK033823 and DK020541 to J.E.P.; DK058498 and DK42583 to C.B.N.
